# Subsoiling increases aggregate-associated organic carbon, dry matter, and maize yield on the North China Plain

**DOI:** 10.7717/peerj.11099

**Published:** 2021-03-24

**Authors:** Ying Shen, Tingting Zhang, Jichao Cui, Siyu Chen, Huifang Han, Tangyuan Ning

**Affiliations:** College of Agronomy, Shandong Agricultural University, Tai’an, Shandong Province, P.R. China

**Keywords:** Aggregate-associated organic carbon, Dry matter, Maize yield, No-tillage, Subsoiling

## Abstract

**Background:**

Soil degradation is one of the main problems in agricultural production and leads to decreases in soil quality and productivity. Improper farming practices speed this process and are therefore not conducive to food security. The North China Plain (NCP) is a key agricultural area that greatly influences food security in China. To explore the effects of different tillage measures on aggregate-associated organic carbon (AOC), the accumulation and transport of dry matter, and maize yield, and to identify the most suitable tillage method for use on the NCP, a field experiment was conducted at Shandong Agricultural University from 2016–2017 using plots that have been farmed using conservation tillage since 2002.

**Methods:**

In this study, Zhengdan 958 summer maize was used as the test material and undisturbed soil and plant samples were obtained under four tillage methods—no-tillage (NT, tillage depth: 0 cm); rotary tillage (RT, tillage depth: 10 cm); conventional tillage (CT, tillage depth: 20 cm); subsoiling (SS, tillage depth: 40 cm)—which were used to determine the AOC and dry matter contents, as well as the yields of two summer maize growing seasons. Each sample was replicated three times and the AOC content was determined via potassium dichromate oxidation colorimetry. Potassium dichromate oxidized organic carbon in organic matter was employed to reduce hexadecent chromium into green trivalent chromium. Colorimetry was then used to determine the amount of reduced trivalent chromium and calculate the organic matter content.

**Results:**

The resulting data were statistically analyzed and the results showed that, compared with CT, the AOC contents with NT and SS increased by 5.65% and 9.73%, respectively, while that with RT decreased by 0.12%. Conventional tillage resulted in the highest mean dry matter weight when the maize reached maturity, which was 19.19%, 9.83%, and 3.38% higher than those achieved using NT, RT, and SS, respectively. No significant difference was found between CT and SS treatments, both of which tended to increase the accumulation of dry matter as well as its contribution of assimilates to grain yield post-anthesis. Compared with CT, the mean yield increased at a rate of 0.18% with SS, while yields declined at rates of 17.17% and 11.15 with NT and RT, respectively. The yield with NT was the lowest, though the harvest indices with NT and SS were higher than those with RT and CT. Overall, SS increased the accumulation of dry matter and its contribution of assimilates to grain yields post-anthesis, as well as the AOC content and yields, making it the ideal tillage method for the NCP.

## Introduction

At present, soil degradation is one of the main environmental problems worldwide (Lasanta, Arnáez & Nadal-Romero, 2019). The deterioration of the soil environment is mainly caused by adverse natural factors and unreasonable land use by humans (Pandit et al., 2018), which leads to declines in soil quality and productivity (Lasanta, Arnáez & Nadal-Romero, 2019). With the growth of Earth’s population, the demand for food will continue to increase, and the decline in productivity caused by soil degradation is not conducive to global food security. Over the past few decades, large areas of soil have been degraded globally, with on- and off-site erosion (Kara et al., 2016) followed by reductions in soil organic carbon (SOC) (Gibbs & Salmon, 2015). Increasing the chelation or accumulation of SOC can effectively improve soil quality, reduce the risk of degradation (Qi et al., 2019), and increase grain yields (Uslu et al., 2020). The components of grain yields depend on and compensate for each other with changes in soil conditions (Xu et al., 2019), and improper tillage measures can change soil conditions, making it vulnerable to severe degradation, resulting in lower yields, and further increasing the risk of food insecurity (Doni et al., 2017).

Conservation tillage is a new tillage method in which straw is returned to the field post-harvest; no-tillage (NT) and less tillage are at the core of this approach (Zhang & Gao, 2005; Li et al., 2020). Conservation tillage systems increased in use from 5.3 to 106 million ha between 1990 and 2009 (Kassam et al., 2009). Research has shown that conservation tillage can improve soil structures (Eden et al., 2020), SOC contents (Guo et al., 2020), and water retention capacities (He et al., 2009; Yang et al., 2020), reduce wind (Gao et al., 2016) and water (Bertol et al., 2003) erosion, and improve the physical properties of soils (Khan et al., 2017). Currently, conservation tillage is widely used around the world, and in some areas of China, NT and subsoiling (SS) are especially common. No-tillage is an effective means of improving soil structures, nutrients, and organic matter (OM) contents (Müller-Nedebock & Chaplot, 2015). Zhang et al. (2015) also found that crop yields increased under NT. Meanwhile, SS can break compacted hardpan layers, reduce soil strengths, improve water use efficiencies (Wang et al., 2020a; Wang et al., 2020b), promote crop growth, and improve crop yields (Jr & Aase, 2003). Some studies have shown that there is a significant positive correlation between maize yield and SOC content (Xu et al., 2019), the latter of which can be directly affected by tillage methods. Reportedly, 90% of SOC exists in the form of aggregates (Karami et al., 2012), and the stability of soil aggregates and SOC contents interact with each other (Liang et al., 2019; Yao et al., 2020). However, the relationship between aggregate-associated organic carbon (AOC) contents and crop yields under different tillage methods has rarely been explored.

Dry matter is the basis of crop yields, as the amount of accumulated dry matter determines crop yields (Xu et al., 2017). Regarding grain yields, improving the production capacity of dry matter, as well as the ability to transfer dry matter to grains post-anthesis, has been effective in improving crop yields (Tollenaar & Daynard, 1982). Yin (2002) also showed that there was a positive correlation between the accumulation of dry matter and crop yields to a certain extent, especially in late growth stages. Tillage practices impact the dry matter of crops, significantly affecting its translocation post-anthesis and contribution to the grain (Shi et al., 2016). Therefore, it is important to study which tillage practices positively affect the accumulation and transport of dry matter to increase crop yields.

The North China Plain (NCP) is an important agricultural area in China, especially for winter wheat and summer maize, which play an important role in national food security. The arable land area of the NCP accounts for 21% of all arable land nationwide, and the grain output of this region accounts for 26% of the national total. The planting area of maize accounts for 31% of all maize grown in China and yields 30% of the national total (China Statistical Yearbook, 2016). Therefore, ensuring increased and stable grain yields in this region is vital to China’s food security.

Conventional tillage (CT) methods on the NCP are practiced using small motorized cultivators equipped with disk plows to loosen the surface soil (0–20 cm), which is known to be unconducive to high and stable grain yields. Long-term CT will not only cause a shallow soil layer but also increase SOC mineralization, soil erosion, and nutrient loss (Feng et al., 2018). In contrast, conservation tillage can reduce the damage caused to the soil by over tillage. The effects of tillage on soils develop over time, while the study of grain yields is complex and benefits cannot be demonstrated in the short term. Therefore, to study the effects of different tillage measures on agricultural soils, we conducted a study in 2016 on a field that has been under conservation tillage since 2002. In this field, four tillage measures were employed—NT, rotary tillage (RT), CT, and SS—with tillage depths are 0, 10, 20, and 40 cm, respectively. The objectives of our study were to: (1) identify ongoing NCP experiments with tillage measures as an experimental factor, (2) analyze the effects of different tillage measures on AOC and plant dry matter, and (3) relate the effects of tillage on crop yields to the experimental data.

## Materials and Methods

### Experimental site

A long-term conservation tillage experiment began in 2002 at the Experimental Station of Shandong Agricultural University (36°10″9′N, 117°9″03′E), which is an area that has a temperate continental climate and exhibits the typical climatic characteristics of the NCP. This area is also characterized by abundant sunlight and distinct seasons, and its mean annual temperature, number of sunshine hours, and rainfall are 13.6 °C, 2,624 h, and 697 mm, respectively. The soil at this site, which is brown loam, has a bulk density of 1.4 g cm^−3^, and it contains 44% silt, 40% sand, and 16% clay. Before the experiment began, the pH of the surface soil was 6.8, and its SOC, total nitrogen, and total phosphorus contents were 6.7, 1.3, and 7.2 g kg^−1^, respectively.

### Crop management and experimental design

The experimental plots were based on a winter wheat–summer maize (*Triticum aestivum* L.–*Zea mays* L.) double-cropping system. The variety of maize was Zhengdan 958, which had a planting density of 66,700 plants/hm^2^. It was sown from June 18–25 every year and harvested from October 8–12 of the same year. During the summer maize growing period, basal fertilizer was applied at a rate of 120 kg N, 120 kg P_2_O_5_, and 100 kg K_2_O ha^−1^, and at the maize joining state, topdressing fertilizer was applied at a rate of 120 kg N ha^−1^ (Lu & Xie, 2011; Fu & Zhang, 2015). The planting specifications and field management strategies employed were the same as those used in generally high-yielding fields. The wheat variety in the experimental field was Jimai 22, which was sown from October 10–15 each year and harvested from June 8–15 of the following year. After the wheat and maize matured, all of the straw was returned to the field.

The experimental field was split into distinct plots, and four tillage measures were adopted—NT (0 cm), RT (10 cm), CT (20 cm), and SS (40 cm). The area of each plot was 30 × 4 m^2^, for which there were three replicates per tillage measure. To minimize the edge effect, a 0.5-m buffer region was set around each plot. The total experimental land was ∼0.16 ha. The winter wheat–summer maize double-cropping system, with tillage measures before the sowing of winter wheat and direct stubble sowing of summer maize, is the main cropping practice in this region.

### Grain sampling and analysis

Samples were collected at the mature stage of maize to determine grain yields. During the experiment, 10-m double-row sampling was used, and the output area was 5 m^2^. From among the harvested ears, 15 were chosen to measure their characteristics after 20 days of natural air drying. The ear characteristics included row number, kernels per row, and kernels per ear. After attaining these measurements, kernels were threshed by a grain thresher, and their 1,000-kernel weights were determined.

### Soil sampling and analysis

During maize harvesting, soil samples at different depths (0–10, 10–20, and 20–40 cm) were collected. To determine the amount of AOC, a composite undisturbed soil sample (homogenized soil from three replicate plots in one treatment) was collected from the different soil layers with the use of a flat spade. Each sample was then placed into an airtight aluminum container and transported to the laboratory. To prevent soil deformation, samples were peeled into soil blocks along their structural textures and soil samples that passed the 1-cm sieve were retained. After the removal of visible organic residue, the samples were air-dried and subjected to wet sieving (Zhang et al., 2019).

Dried soil (100 g) was weighed and placed on an aggregate analyzer (TTF-100, Shangyu Shunlong Laboratory Instruments, China). The pore sizes of the sieves were 5 mm, 2 mm, 0.25 mm, and 0.053 mm from the top to the bottom (Cambardella & Elliott, 1993). Deionized water was slowly poured into the sieve barrel until it was level with the edge. Soil samples were first infiltrated for 10 min and then the amplitude of the agglomerate analyzer was adjusted to 20 times/min and screened for 10 min. After screening, the aggregates on each sieve were washed into an aluminum box with deionized water and let to stand for 48 h. The supernatant was then discarded from the aluminum box and the samples were left to air dry naturally. The AOC contents of air-dried aggregates were then determined via potassium dichromate external heating (Bao, 2000). Each analysis was repeated three times.

### Plant sampling and analysis

In the middle of each plot, five successive uniform plants were selected manually and cut at ground level at anthesis and physiological maturity. The plants were packed into different envelopes according to their different organs and then dried to a constant weight at 70 °C to determine the dry matter content. Dry matter accumulation and distribution indices were calculated using the following equations (Ma et al., 2015b):

(1) \begin{eqnarray*}\text{Translocation}\nonumber\\\displaystyle \qquad =\text{dry matter at anthesis}-\text{dry matter of vegetative plant parts at maturity}\end{eqnarray*}

(2) \begin{eqnarray*}\text{Translocation efficiency}\nonumber\\\displaystyle \qquad =(\text{dry matter translocation/dry matter at anthesis})\times 100\end{eqnarray*}

(3) \begin{eqnarray*}\text{Contribution of pre-anthesis assimilates to grain}\nonumber\\\displaystyle \qquad =(\text{translocation/grain yield})\times 100\end{eqnarray*}

(4) \begin{eqnarray*}\text{Accumulation post-anthesis}\nonumber\\\displaystyle \qquad =\text{dry matter of grains at maturity}-\text{dry matter translocation}\end{eqnarray*}

(5) \begin{eqnarray*}\text{Contribution of accumulation post-anthesis to grains}\nonumber\\\displaystyle \qquad =(\text{accumulation post-anthesis/dry matter of grains at maturity})\times 100\end{eqnarray*}

(6) \begin{eqnarray*}\text{Harvest index (HI)}=\text{grain yield/total aboveground biomass at maturity}.\end{eqnarray*}

### Data processing

An analysis of variance (ANOVA) was used according to a 2 × 4 factorial design with three replications per treatment to assess the soils and crop yields of each experimental treatment. Simultaneously, the least significant difference (LSD) was used for multiple comparisons. The significance level for the testing of all hypotheses was preset *at p* < 0.05. Pearson’s correlation coefficients were used to analyze the correlations between variables. All statistical analyses were performed using SPSS v. 19.0 (SPSS Inc., USA) (Xue, 2019) and Microsoft Excel v. 2016 (Microsoft Corp., USA).

## Results

### Yield components, yields, and harvest indices

The yields and components of summer maize in 2016 and 2017 are shown in Table 1. The lowest productive ear number appeared under the SS treatment in 2017, but there was no significant difference among the treatments. The number of grains per ear was the lowest in the CT treatment in 2017, and the highest in the SS treatment, the latter of which was significantly higher than that in the CT treatment. There was no significant difference between the CT, RT, and NT treatments. In terms of 1000-grain weight and grain yield, the order of each treatment was SS > CT > RT > NT. In terms of yield, the difference between SS and CT was not significant, but their yields were significantly higher than those with RT and NT. Compared with CT, the mean yield increased at a rate of 0.18% with SS, while yields declined at rates of 17.17% and 11.15% with NT and RT, respectively. The yield of the NT treatment was the lowest, but the HI indices with NT and SS were higher than those with RT or CT.

**Table 1:** Effects of tillage on maize yield and its components.

**Year**	**Treatment**	**Productive ear No. (10^4^/ha)**	**Grain per ear (grain/ear)**	**1,000–grain weight (g)**	**Grain yield (Mg/ha)**	**HI**
2016	NT	6.67^a^	531.18^bc^	302.00^c^	11.46^c^	0.481^a^
	RT	6.67^a^	556.81^ab^	311.76^bc^	12.71^b^	0.471^b^
	CT	6.66^a^	552.57^ab^	311.89^bc^	13.55^a^	0.463^c^
	SS	6.66^a^	564.66^a^	328.27^a^	13.57^a^	0.479^a^
2017	NT	6.69^a^	581.26^ab^	326.86^c^	10.97^b^	0.468^b^
	RT	6.71^a^	553.30^ab^	335.29^b^	11.35^b^	0.442^c^
	CT	6.70^a^	520.01^b^	343.67^a^	13.53^a^	0.464^b^
	SS	6.58^a^	595.84^a^	345.50^a^	13.56^a^	0.482^a^

**Notes.**

Different letters in each column indicate significant differences between different tillage measures (*P*<0.05; Duncan’s test).

NT no-tillage RT rotary tillage CT conventional tillage SS subsoiling HI harvest index

### Aggregate-associated Organic Carbon (AOC)

**Figure 1:**
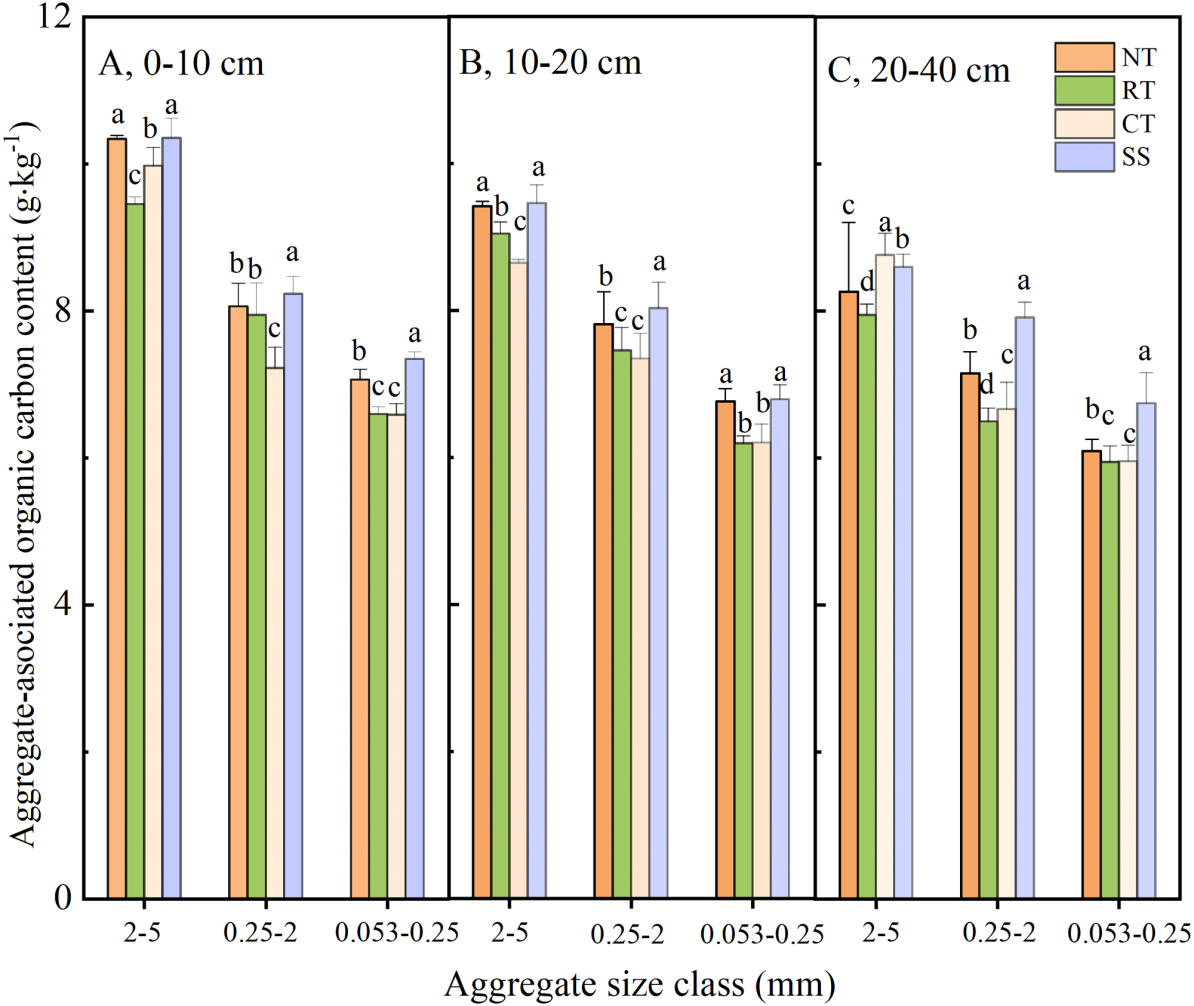
Aggregate-associated organic carbon under different soil layers in different treatments. NT (no-tillage), RT (rotary tillage), CT (conventional tillage), SS (subsoiling). The different letters in the picture indicate that they are significantly different in different treatments at the 5% level. Vertical bars are standard errors.

The AOC contents of the experimental soils are shown in Fig. 1. Among all aggregates, the highest AOC content was found in 2–5-mm aggregates at soil depths of 0–10 cm. At the same soil depth, the AOC contents decreased with decreases in particle sizes (i.e., 2–5 mm > 0.25–2 mm > 0.25–0.053 mm). From the perspective of different tillage methods, the AOC content with SS was the highest among all treatments, except for aggregates with 2–5 mm particle sizes under 20–40 cm of soil. Secondly, the AOC content in the NT treatment was significantly higher than those with RT or CT once 2–5-mm particle aggregates were removed from the 20–40-cm soil layer. The AOC content at particle sizes of 2–5 mm and soil depths of 20–40 cm were the highest in the CT treatment. Compared with CT, the AOC content with NT, RT, and SS changed by +5.65%, −0.12%, and +9.73%, respectively.

### Accumulation, partitioning, and translocation of dry matter

Tillage measures had a significant impact on the indicators described in Table 2. Except for the dry matter of vegetative plant parts at maturity, growth years and the interaction of growth years and tillage measures significantly influenced the other indices. The dry matter content of maize under different tillage measures is shown in Table 3. In the anthesis stage, the contents of leaves and culm and the total dry matter weight of each tillage treatment were ordered as follows: CT > SS > RT > NT. Compared with CT, the NT, RT, and SS treatments reduced the leaf and culm dry matter weight in the anthesis period by 11.44%, 7.47%, and 3.48%, and the total dry matter weight during anthesis by 10.85%, 10.01%, and 3.87%, respectively, over two years. The order of ear weights in the anthesis period was as follows: CT > SS > NT > RT. Compared with CT, the NT, RT and SS treatments reduced the ear dry matter weight of maize in the anthesis period by 8.41%, 19.16%, and 5.14%, respectively, over two years. The weight distribution of mature vegetative plant parts was the same as that of the total dry matter weight during anthesis, and the total dry matter weight at maturity was also the highest with CT. Compared with CT, the NT, RT, and SS treatments reduced the dry weights of mature vegetative plant parts by 20.95%, 8.72%, and 6.47%, and their associated total dry matter weights decreased by 19.19%, 9.83%, and 3.38%, respectively. The dry matter weight of grains was maximized with SS at maturity, and was significantly higher than those with NT and RT, though there was no significant difference between SS and CT. Compared with CT, the NT, RT, and SS treatments changed the grain dry matter weight at maturity by −17.15%, −11.11%, and +0.20%, respectively.

**Table 2:** Analysis of variance of dry matter and dry matter translocation indices that were affected by growth year and tillage measures.

	**Anthesis**			**Maturity**			**DMT**	**DMTE**	**CDMRG**
	**Leaf + culm**	**Ear**	**Total**	**Vegetative plant part**	**Grain**	**Total**			
Tillage (T)	^***^	^***^	^***^	^***^	^***^	^***^	^***^	^***^	^***^
Year (Y)	^**^	^***^	^***^	ns	^***^	^***^	^**^	^**^	^***^
Y × T	^**^	^***^	^**^	ns	^***^	^***^	^**^	^*^	^**^

**Notes.**

DMT dry matter translocation DMTE dry matter translocation efficiency CDMRG contribution of pre-anthesis assimilates to grain (%) ns not significant

* *P*<0.05.

** *P*<0.01.

*** *P*<0.001.

**Table 3:** Effects of tillage on the dry matter contents of vegetative and reproductive parts in anthesis and maturity of maize.

	**Treatments**	**Anthesis**			**Maturity**		
		**Leaf + culm**	**Ear**	**Total**	**Vegetative plant part**	**Grain**	**Total**
2016	NT	243.6^d^	70.2^c^	313.8^c^	185.1^d^	171.8^c^	356.9^c^
	RT	255.3^c^	60.5^d^	315.8^c^	214.0^c^	190.6^b^	404.6^b^
	CT	283.9^a^	77.2^a^	361.1^a^	235.7^a^	203.1^a^	438.8^a^
	SS	270.8^b^	74.4^b^	345.2^b^	221.7^b^	203.4^a^	425.1^a^
2017	NT	245.9^c^	67.0^b^	312.9^c^	186.6^c^	164.5^b^	351.1^d^
	RT	256.2^b^	60.6^c^	316.8^c^	215.2^b^	170.2^b^	385.4^c^
	CT	269.3^a^	72.6^a^	341.9^a^	234.5^a^	202.8^a^	437.3^a^
	SS	263.0^ab^	67.7^b^	330.6^b^	218.1^b^	203.3^a^	421.4^b^

**Notes.**

Different letters in each column indicate significant differences between different tillage measures (*P* <0.05; Duncan’s test).

NT no-tillage RT rotary tillage CT conventional tillage SS subsoiling

Shi et al. (2016) showed that tillage has an effect on the transportation of dry matter and its contribution to the grain yields of wheat—similar to the results presented here. In this study, the translocation and translocation efficiency of dry matter with CT and RT were lower than those with NT and SS (Table 4). No-tillage increased the accumulation of pre-anthesis dry matter and its contribution of assimilates to grain yields. At the same time, CT and SS tended to increase the accumulation of dry matter, as well as its contribution of assimilates to grain yields post-anthesis. This may be because different tillage methods have different effects on the soil environment, thus affecting the growth, development, and yield of crops (Bisheng et al., 2015).

**Table 4:** Effects of tillage on dry matter transfer efficiency.

**Treatments**		**Pre-anthesis dry matter accumulated**			**Post-anthesis dry matter accumulated**	
		Dry matter transl. (kg/hm^2^)	Dry matter transl. efficiency (%)	Contr. assimilates to grain (%)	Accumulation into grain (kg/hm^2^)	Contr. assimilates to grain (%)
2016	NT	3896.10^a^	24.01^a^	34.05^a^	7546.72^c^	65.95^b^
	RT	2750.58^c^	16.18^d^	21.67^c^	9940.36^b^	78.33^a^
	CT	3210.12^b^	16.98^c^	23.73^b^	10320.00^a^	76.27^a^
	SS	3270.06^b^	18.13^b^	24.13^b^	10280.00^ab^	75.87^a^
2017	NT	3949.38^a^	24.12^a^	36.06^a^	7004.17^d^	63.94^c^
	RT	2730.60^c^	16.00^c^	24.09^b^	8602.38^c^	75.91^b^
	CT	2317.68^d^	12.92^d^	17.16^d^	11192.04^a^	82.84^a^
	SS	2990.34^b^	17.07^b^	22.09^c^	10549.33^b^	77.91^b^

**Notes.**

Different letters in each column indicate significant differences between different tillage measures (*P* <0.05; Duncan’s test).

NT no-tillage RT rotary tillage CT conventional tillage SS subsoiling

### Correlations

The experimental correlations among crop yields, physiological characteristics, and AOC contents are shown in Table 5. Maize yield was significantly and positively correlated with the translocation and translocation efficiency of dry matter, as well as with AOC_1_, AOC_2_, and AOC_3_ contents. Unsurprisingly, the translocation of dry matter displayed a significant positive correlation with its translocation efficiency, as well as with the contribution of pre-anthesis assimilates to grains, AOC_1_, and AOC_2_. The dry matter translocation efficiency was also significantly and positively correlated with the contribution of pre-anthesis assimilates to grains, AOC_1_, and AOC_2_, and the contribution of pre-anthesis assimilates to grains was significantly and positive correlated with AOC_2_. Finally, AOC_1_ showed significant positive correlations with AOC_2_ and AOC_3_, and AOC_2_ was significantly and positively correlated with AOC_3_.

**Table 5:** Pearson correlation coefficient of the annual yield, physiological characteristics and aggregate-associated organic carbon content in 2016 and 2017.

	1	2	3	4	5	6	7
1 Grain yield	–						
2 Dry matter transl.	0.540^**^	–					
3 Dry matter transl. efficiency (%)	0.409^*^	0.989^***^	–				
4 Contr. of preanth. assimilates to grain (%)	−1.70	0.731^***^	0.822^***^	–			
5 AOC_1_	0.694^***^	0.707^***^	0.640^**^	0.310	–		
6 AOC_2_	0.609^**^	0.730^***^	0.680^***^	0.405^*^	0.993^***^	–	
7 AOC_3_	0.832^***^	0.285	0.160	−0.301	0.812^***^	0.742^***^	–

**Notes.**

AOC_1_, AOC_2_ and AOC_3_ represent the content of aggregate-associated organic carbon in 0–10, 10–20 and 20–40 cm soil layers respectively.

* *P* <0.05.

** *P*<0.01.

*** *P*<0.001.

## Discussion

### Effects of different tillage methods on maize yield and its components

High crop yield is the most important purpose of agricultural production; thus, it is important to consider the effect of tillage measures on crop yields. In some studies, it has been demonstrated that conservation tillage can improve soil fertility (Wang et al., 2019), maintain maize yield (Shao et al., 2016), and guarantee increased production (Ren et al., 2016). In this study, conducted from 2016–2017, SS resulted in a higher maize yield than other treatments and had a positive effect on the number of grains per ear and 1,000-grain weight, similar to the findings of Kuang et al. (2020). At the same time, the AOC content in the SS treatment was also higher than in other treatments. Lal (2009) found that there was a close relationship between yields and SOC. Soil organic carbon is an important soil characteristic and plays an important role in soil fertility and sustainable agricultural development (Su & Zhao, 2002; Li et al., 2020). It is considered to be a decisive factor affecting soil fertility and crop yields (Qiu et al., 2009). In this study, SS increased the organic carbon content in soil aggregates and soil fertility and promoted increases in crop yield (Xie et al., 2020). Subsoiling can not only increase soil AOC and improve soil fertility, but also break hard plow bottoms (Ma et al., 2015b), improve soil pore conditions, and increase permeability (Wang et al., 2007), all of which are conducive to the distribution of plant roots in deep soils (Sun et al., 2017) and promote the use of nutrients and water therein. Thus, SS is conducive to high crop yields.

In this study, the AOC content with NT was only lower than that with SS, which was better than the contents in the RT and CT treatments. Considering the NT measure employed, its effect on the environment was insignificant (Basso et al., 2011), and may result in a decrease in organic carbon mineralization (Kan et al., 2020b), though it remains an important conservation tillage approach. Nevertheless, the results of studies regarding its effect on maize yield in China and abroad have not been consistent. Some studies had shown that NT had a positive effect on yield; for example, Lamm, Aiken & Kheira (2009) showed that NT could effectively increase crop yields. However, Wang & Li (2014) showed that in the arid lands of northern China, continuous NT reduced crop yields by 12–18%. These differences may be attributable to different soil types and climatic conditions, which led to NT affecting yields differently between the regions. It is also possible that the length of time the NT process takes differs by region (Boomsma et al., 2010). Wang et al. (2012) showed that NT was beneficial to increasing the contents of soil aggregates, thereby increasing soil nutrients; however, it was suggested that years of NT would increase soil compactness and reduce crop yields. Here, NT resulted in the lowest maize yield and it was found that long-term NT could cause soil hardening and increases in bulk density (Kan et al., 2020a), affecting the development of crop root systems, as well as nutrient and water absorption, which may be why the lowest yields were recorded with NT in this study (Ren et al., 2018).

Conventional tillage showed a certain advantage with respect to maize yield, which was significantly higher than those with RT and NT. Under the conditions of NT and RT, the volumetric weight of the soil was large and the plow bottoms remained hard (Hua, Guo & Zhang, 2008; Gong & Lv, 2014; Ma et al., 2014), while CT greatly disturbed the soil, which can break the plow bottom to a certain extent and increase the distribution of the root systems in deep soils (Zhai et al., 2017; Wang et al., 2020b), both of which are conducive to increases in crop yield. Nevertheless, while the yield of the CT treatment was high, the HI was significantly lower than those of the NT and SS treatments. The HI is an important index for evaluating yields and cultivation is significantly correlated with economic yield. In this study, the HI was the greatest in the SS and NT treatments. Although the maize yield and dry matter weights with NT were the lowest, the HI was high, and while the CT treatment was better than NT in terms of grain yield, it did not exhibit any advantages in HI over NT. This may be because CT was more helpful that NT for improving the dry matter weight rather than the grain weight of maize plants.

### Effects of different tillage measures on AOC

The differences in AOC contents at different soil depths are mainly caused by the surface layer receiving external OM and the transformation and exchange of SOC in this layer. Newly input OM first accumulates and decomposes on the soil surface, and then infiltrates into the deep soil (Wei et al., 2015). By turning the soil during tillage, OM on the surface layer can be transported to deeper soil layers, causing differences in the AOC contents of different soil layers. In this study, compared with CT and RT, NT primarily increased AOC content in the 0–20-cm soil layer. Instead of soil turnover, NT reduced the exposed area, minimized soil disturbance, and reduced the mineralization rate of organic carbon (Chen et al., 2013), which resulted in increases in AOC due to long-term accumulation. Subsoiling significantly increased AOC content in the 0–40-cm soil layer, as previously described by Chan, Heenan & Oates (2002). Furthermore, Liu (2019) showed that SS could reduce soil compaction and bulk density (Zhang et al., 2017), and promote the transformation of straw, stubble, and roots into deep soils, thus increasing the carbon contents of deep soils. Kushwa et al. (2016) found that CT often interferes with soil aggregates, leading to the exposure of the organic carbon protected by them, as well as the turnover of macro-aggregates, which leads to the decomposition of soil aggregates and AOC loss and limits crop yields. Compared with CT, the mean AOC content in the 0–40-cm soil layer increased by 5.4% and 9.1%, respectively, with NT and SS, but there was no significant difference between RT and CT. Meanwhile, the AOC content decreased with decreasing aggregate size (i.e., 2–5 mm > 0.25–2 mm > 0.053–0.25 mm). Carter (1992) also showed that aggregate particle size was positively correlated with AOC content and Huo et al. (2019) found that conservation tillage contributed more to the formation of macro-aggregates and increases in AOC. The results of this study show that there is a significantly positive correlation between AOC content and maize yield, and this correlation was strongest at soil depths of 20–40 cm. When there are more macro-aggregates in the soil, more AOC will be available, favoring higher crop yields.

### Accumulation, partitioning, and remobilization of dry matter

Dry matter is the highest form of photosynthetic products in crops, and its accumulation is closely related to grain yield. The accumulation of crop dry matter is restricted by many factors, such as tillage practices (Chen et al., 2014) and fertilization systems. Different tillage methods have different effects on the accumulation and distribution of dry matter. Among them, tillage practices can regulate accumulation by changing the hydrothermal characteristics of the soil (Yin et al., 2016). In this study, the dry matter weight of maize with CT was the greatest. Guan et al. (2014) also showed that after NT, short-term CT significantly increased the aboveground biomass of NCP maize, similar to our findings. In addition to the CT treatment, the dry matter weight of the SS treatment was also high, and Huang et al. (2009) found that the increase in dry matter was mainly due to improved photosynthesis.

Subsoiling can delay the senescence of maize leaves, thus maintaining a higher leaf area index and photosynthetic rate, which favor increased maize dry matter weights and lay a solid physiological foundation for an overall increase in grain yields (Sun et al., 2017). Additionally, Shi et al. (2016) showed that tillage methods effect the accumulation, transport, and contribution of stem dry matter to grain yields during the late anthesis stage in wheat. In this study, NT increased the pre-anthesis accumulation of dry matter and its contribution to grain yields, while CT and SS tended to increase the accumulation of dry matter, as well as its contribution to gain yields post-anthesis. This may be because different tillage methods have different effects on the soil environment, and thus affect the growth, development, and yields of crops (Bisheng et al., 2015).

Crop yield was determined by the pre-anthesis accumulation of carbohydrates and the transport to grains post-anthesis (Jiang et al., 2002). The pre-anthesis accumulation of dry matter and the transport to grains post-anthesis of maize with SS and CT were higher than those of the NT treatments, which provided a foundation for increases in yield. Moreover, past studies have shown that CT and SS can improve water use efficiency (Li et al., 2006), promote chlorophyll synthesis, and slow chlorophyll degradation, thus prolonging the functional period and increasing the distribution of dry matter post-anthesis, thereby improving the growth rate, ensuring the accumulation of dry matter and grain filling, and ultimately leading to increased crop yields (Xu et al., 2017).

## Conclusions

In summary, our results from two years of field experiments showed that SS effectively increased the AOC content, with the effect being better than in the other three treatments. Although the dry matter content was slightly lower than that of the CT treatment, the difference between SS and CT was not significant in this regard. The yield with SS was the highest among the four tillage methods, and this method of tillage also effectively improved the HI. No-tillage also increased the AOC content and HI, but the dry matter contents and yields of the NT treatment were the lowest. Conventional tillage resulted in more dry matter content at maturity but displayed no advantage with respect to AOC content, yield, or HI when compared to SS or NT. Finally, RT did not show any significant advantages in this study. Overall, among the research indices involved in this study, SS was found to be the ideal tillage method. It is essential to study the effects of conservation tillage on cultivated lands in order to improve grain yields and ensure national food security on the NCP, and this study provides guidance for optimizing agricultural tillage management in this vital agricultural region.

##  Supplemental Information

10.7717/peerj.11099/supp-1 Data S1 Data measured in this test

## Additional Information and Declarations

### Data Availability

The following information was supplied regarding data availability: Raw measurements are available as Supplemental File.

## References

[ref-1] Bao SD (2000). Soil agrochemical analysis.

[ref-2] Basso B, Sartori L, Bertocco M, Cammarano D, Martin EC, Grace PR (2011). Economic and environmental evaluation of site-specific tillage in a maize crop in NE Italy. European Journal of Agronomy.

[ref-3] Bertol I, Lemos ME, Cláudio GJ, Vedana ZAL, Roberto CM (2003). Nutrient losses by water erosion. Scientia Agricola.

[ref-4] Bisheng W, Dianxiong C, Xueping WU, Jing LI, Guopeng L, Weishui YU, Xiangling W, Yiyu Y, Xiaobin W (2015). Effects of long-term conservation tillage on soil organic carbon, maize yield and water utilization. Journal of Plant Nutrition & Fertilizer.

[ref-5] Boomsma CR, Santini JB, West TD, Brewer JC, McIntyre LM, Vyn TJ (2010). Maize grain yield responses to plant height variability resulting from crop rotation and tillage system in a long-term experiment. Soil & Tillage Research.

[ref-6] Cambardella CA, Elliott ET (1993). Carbon and nitrogen distribution in aggregates from cultivated and native grassland soils. Soil Science Society of America Journal.

[ref-7] Carter MR (1992). Influence of reduced tillage systems on organic matter, microbial biomass, macro-aggregate distribution and structural stability of the surface soil in a humid climate. Soil & Tillage Research.

[ref-8] Chan KY, Heenan DP, Oates A (2002). Soil carbon fractions and relationship to soil quality under different tillage and stubble management. Soil & Tillage Research.

[ref-9] Chen W, Deng XP, Eneji AE, Wang LL, Xu Y, Cheng YJ (2014). Dry-matter partitioning across parts of the wheat internode during the grain filling period as influenced by fertilizer and tillage treatments. Communications in Soil Science and Plant Analysis.

[ref-10] Chen XW, Shi XH, Zhang XP, Liang AZ, Jia SX, Fan RQ, Wei SC (2013). Evaluating tillage practices impacts on soil organic carbon based on least limiting water range. Acta Ecologica Sinica.

[ref-11] Doni S, Macci C, Vincenzo L, Aymen S, Carlos G, Grazia M (2017). Innovative system for biochemical monitoring of degraded soils restoration. Catena An Interdisciplinary Journal of Soil Science Hydrology Geomorphology Focusing on Geoecology & Landscape Evolutionm.

[ref-12] Eden M, Bachmann J, Cavalaris C, Kostopoulou S, Kozaiti M, Böttcher J (2020). Soil structure of a clay loam as affected by long-term tillage and residue management. Soil & Tillage Research.

[ref-13] Feng QQ, Han HF, Zhang Y, Xu J, Cao YQ, Wang SB, Ning TY, li ZJ (2018). Effects of tillage methods on soil carbon sequestration and water holding capacity and yield in wheat-maize rotation. Journal of Plant Nutrition & Fertilizers.

[ref-14] Fu RY, Zhang MG (2015). A practical method of soil testing and formulated fertilization for crops–target yield method, taking maize as an example. Information of Agricultural Science and Technology.

[ref-15] Gao Y, Dang X, Yu Y, Li Y, Liu Y (2016). Effects of tillage methods on soil carbon and wind erosion. Land Degradation & Development.

[ref-16] Gibbs HK, Salmon JM (2015). Mapping the world’s degraded lands. Applied Geography.

[ref-17] Gong DQ, Lv J (2014). Effects of soil texture on variations of paddy soil physical and chemical properties under continuous no tillage. Acta Ecologica Sinica.

[ref-18] Guan D, Al-Kaisi MM, Zhang Y, Duan L, Tan W, Zhang M, Li Z (2014). Tillage practices affect biomass and grain yield through regulating root growth, root-bleeding sap and nutrients uptake in summer maize. Field Crops Research.

[ref-19] Guo YF, Fan RQ, Zhang XP, Zhang Y, Wu GH, McLaughlin N, Zhang SX, Chen XW, Jia SX, Liang AZ (2020). Tillage-induced effects on SOC through changes in aggregate stability and soil pore structure. Science of The Total Environment.

[ref-20] He J, Kuhn NJ, Zhang XM, Zhang XR, Li HW (2009). Effects of 10 years of conservation tillage on soil properties and productivity in the farming-pastoral ecotone of Inner Mongolia, China. Soil Use & Management.

[ref-21] Hua WD, Guo YF, Zhang ZX (2008). Influence of plough pan on broke partially slope farmland to moisture content infiltration. Journal of Soil & Water Conservation.

[ref-22] Huang M, Wu J, Li Y, Yao Y, Jin K (2009). Effects of different tillage managements on production and yield of winter wheat in dryland. Transactions of the Chinese Society of Agricultural Engineering.

[ref-23] Huo L, Yang SC, Wang CB, Jiang WL, Wen MJ (2019). Effects of tillage types on soil aggregate distribution and stability in irrigated sierozem of Gansu Yellow River irrigation area, China. Chinese Journal of Applied Ecology.

[ref-24] Jiang D, Yu ZW, Li YG, Yu SL (2002). Effects of different nitrogen application levels on changes of sucrose content in leaf, culm, grain and photosynthate distribution and grain starch accumulation of winter wheat. Scientia Agricultura Sinica.

[ref-25] Jr JLP, Aase JK (2003). Water Infiltration and storage affected by subsoiling and subsequent tillage. Soilence Society of America Journal.

[ref-26] Kan ZR, Liu QY, He C, Jing ZH, Virk AL, Qi JY, Zhao X, Zhang HL (2020a). Responses of grain yield and water use efficiency of winter wheat to tillage in the North China Plain. Field Crops Research.

[ref-27] Kan ZR, Virk AL, He C, Liu QY, Qi JY, Dang YP, Zhao X, Zhang HL (2020b). Characteristics of carbon mineralization and accumulation under long-term conservation tillage. Catena.

[ref-28] Kara O, Babur E, Altun L, Seyis M (2016). Effects of afforestation on microbial biomass C and respiration in eroded soils of Turkey. Journal of Sustainable Forestry.

[ref-29] Karami A, Homaee M, Afzalinia S, Ruhipour H, Basirat S (2012). Organic resource management: impacts on soil aggregate stability and other soil physico-chemical properties. Agriculture, Ecosystems & Environment.

[ref-30] Kassam A, Friedrich T, Shaxson F, Pretty J (2009). The spread of conservation agriculture: justification, sustainability and uptake. International Journal of Agricultural Sustainability.

[ref-31] Khan S, Shah A, Nawaz M, Khan M (2017). Impact of different tillage practices on soil physical properties, nitrate leaching and yield attributes of maize (*Zea mays* L.). Journal of Soil Science & Plant Nutrition.

[ref-32] Kuang N, Tan D, Li H, Gou Q, Li Q, Han H (2020). Effects of subsoiling before winter wheat on water consumption characteristics and yield of summer maize on the North China Plain. Agricultural Water Management.

[ref-33] Kushwa V, Hati KM, Sinha NK, Singh RK, Mohanty M, Somasundaram J, Jain RC, Chaudhary RS, Biswas AK, Patra AK (2016). Long-term conservation tillage effect on soil organic carbon and available phosphorous content in vertisols of central India. Agricultural Research.

[ref-34] Lal R (2009). Soils and food sufficiency. A review. Agronomy for Sustainable Development.

[ref-35] Lamm FR, Aiken RM, Kheira AAA (2009). Corn yield and water use characteristics as affected by tillage. Plant Density, and Irrigation. Transactions of the Asabe.

[ref-36] Lasanta T, Arnáez J, Nadal-Romero E, Pereira P (2019). Chapter three—soil degradation, restoration and management in abandoned and afforested lands. Advances in Chemical Pollution, Environmental Management & Protection.

[ref-37] Li Y, Li Z, Chang SX, Cui S, Jagadamma S, Zhang QP, Cai YJ (2020). Residue retention promotes soil carbon accumulation in minimum tillage systems: implications for conservation agriculture. Science of The Total Environment.

[ref-38] Li Y, Wu J, Huang M, Yao Y, Jin K (2006). Effects of different tillage systems on photosynthesis characteristics of flag leaf and water use efficiency in winter wheat. Transactions of the Chinese Society of Agricultural Engineering.

[ref-39] Liang A, Zhang Y, Zhang X, Yang X, McLaughlin N, Chen X, Guo Y, Jia S, Zhang S, Wang L, Tang J (2019). Investigations of relationships among aggregate pore structure, microbial biomass, and soil organic carbon in a Mollisol using combined non-destructive measurements and phospholipid fatty acid analysis. Soil & Tillage Research.

[ref-40] Liu Z (2019). Effects of tillage and straw mulching on soil carbon sources and photosynthetic carbon capture of crops. D. Phil. Thesis.

[ref-41] Lu X, Xie YH (2011). Soil fertilizer science.

[ref-42] Ma Q, Yu WT, He MH, Zhou H, JIiang CM, Xu YG (2014). Effects of subsoiling on soil properties of farmland and corn yield in Luvisols. Chinese Journal of Soil Science.

[ref-43] Ma S, Yu Z, Shi Y, Gao Z, Luo L, Chu P, Guo Z (2015a). Soil water use, grain yield and water use efficiency of winter wheat in a long-term study of tillage practices and supplemental irrigation on the North China Plain. Agricultural Water Management.

[ref-44] Ma S, Yu Z, Shi Y, Zhang Y, Zhao J (2015b). Effect of field border width for irrigation on dry matter accumulation and distribution, yield, and water use efficiency of wheat. Acta Ecologica Sinica.

[ref-45] Müller-Nedebock D, Chaplot V (2015). Soil carbon losses by sheet erosion: a potentially critical contribution to the global carbon cycle. Earth Surface Processes & Landforms.

[ref-46] Pandit R, Scholes R, Montanarella L, Brainich A, Barger N, Brink B, Cantele M, Erasmus B, Fisher J, Gardner T (2018). Summary for policymakers of the assessment report on land degradation and restoration of the intergovernmental science-policy platform on biodiversity and ecosystem services.

[ref-47] Qi JY, Wang X, Zhao X, Pu C, Hi Zhang (2019). Temporal variability of soil organic carbon in paddies during 13-year conservation tillage. Land Degradation & Development.

[ref-48] Qiu JG, Wang LG, Li H, Tang HJ, Ranst EV (2009). Modeling the impacts of soil organic carbon content of croplands on crop yields in China. Scientia Agricultura Sinica.

[ref-49] Ren BZ, Li X, Dong ST, Liu P, Zhao B, Zhang JW (2018). Soil physical properties and maize root growth under different tillage systems in the North China Plain. The Crop Journal.

[ref-50] Ren X, Zhang P, Chen X, Guo J, Jia Z (2016). Effect of different mulches under rainfall concentration system on corn production in the semi-arid areas of the loess plateau. Scientific Reports.

[ref-51] Shao YH, Xie YX, Wang CY, Yue JQ, Yao YQ, Li XD, Liu WX, Zhu YJ, Guo TC (2016). Effects of different soil conservation tillage approaches on soil nutrients, water use and wheat-maize yield in rainfed dry-land regions of North China. European Journal of Agronomy.

[ref-52] Shi Y, Yu Z, Man J, Ma S, Gao Z, Zhang Y (2016). Tillage practices affect dry matter accumulation and grain yield in winter wheat in the North China Plain. Soil & Tillage Research.

[ref-53] State Statistical Bureau (2016). China statistical yearbook.

[ref-54] Su YZ, Zhao HL (2002). Advances in researches on soil organic carbon storages, affecting factors and its environmental effects. Journal of Desert Research.

[ref-55] Sun X, Ding Z, Wang X, Hou H, Zhao M (2017). Subsoiling practices change root distribution and increase post-anthesis dry matter accumulation and yield in summer maize. PLOS ONE.

[ref-56] Tollenaar M, Daynard TB (1982). Effect of source–sink ratio on dry matter accumulation and leaf senesence of maize. Canadian Journal of Plant Science.

[ref-57] Uslu OS, Babur E, Alma MH, Solaiman ZM (2020). Walnut shell biochar increases seed germination and early growth of seedlings of fodder crops. Agriculture.

[ref-58] Wang XB, Cai DX, Hoogmoed WB, Oenema O, Perdok UD (2007). Developments in conservation tillage in rainfed regions of North China. Soil & Tillage Research.

[ref-59] Wang YX, Chen SP, Zhang DX, Yang L, Cui T, Jing HR, Li YH (2020b). Effects of subsoiling depth, period interval and combined tillage practice on soil properties and yield in the Huang-Huai-Hai Plain, China. Journal of Integrative Agriculture.

[ref-60] Wang YL, Li J (2014). Study of tillage patterns suitable for soil physicochemica properties and crop yields in wheat /maize fields. Journal of Plant Nutrition & Fertilizer.

[ref-61] Wang X, Qi JY, Zhang XZ, Li SS, AhmadLatif V, Zhao X, Xiao XP, Zhang HL (2019). Effects of tillage and residue management on soil aggregates and associated carbon storage in a double paddy cropping system. Soil & Tillage Research.

[ref-62] Wang SL, Wang H, Hafeez MB, Zhang Q, Yu Q, Wang R, Wang X, Li J (2020a). No-tillage and subsoiling increased maize yields and soil water storage under varied rainfall distribution: a 9-year site-specific study in a semi-arid environment. Field Crops Research.

[ref-63] Wang HG, Yu ZW, Zhang YL, Wang D (2012). Effects of tillage regimes on water consumption and dry matter accumulation in dryland wheat. Acta Agronomica Sinica.

[ref-64] Wei XR, Gao JL, Zhang XC, Qiu LP (2015). Dynamics of soil aggregate-associated organic carbon along an afforestation chronosequence. Plant & Soil.

[ref-65] Xie HJ, Wang LL, Li LL, Coulter JA, Chai Q, Zhang RZ, Luo ZZ, Carberry P, Rao KPC (2020). Subsoiling increases grain yield, water use efficiency, and economic return of maize under a fully mulched ridge-furrow system in a semiarid environment in China. Soil & Tillage Research.

[ref-66] Xu J, Han H, Ning T, Li Z, Lal R (2019). Long-term effects of tillage and straw management on soil organic carbon, crop yield, and yield stability in a wheat-maize system. Field Crops Research.

[ref-67] Xu J, He ZK, Feng QQ, Zhang YY, Li XS, Xu JJ, Lin X, Han HF, Ning TY, Li ZJ (2017). Effect of tillage method on photosynthetic characteristics and annual yield formation of winter wheat-summer maize cropping system. Plant Nutrition & Fertilizer Science.

[ref-68] Xue W (2019). Data analysis based on SPSS.

[ref-69] Yang HK, Wu G, Mo P, Chen SH, Wang SY, Xiao Y, Ma HL, Wen T, Guo X, Fan GQ (2020). The combined effects of maize straw mulch and no-tillage on grain yield and water and nitrogen use efficiency of dry-land winter wheat (*Triticum aestivum* L.). Soil & Tillage Research.

[ref-70] Yao Y, Liu J, Wang Z, Wei X, Zhu H, Fu W, Shao M (2020). Responses of soil aggregate stability, erodibility and nutrient enrichment to simulated extreme heavy rainfall. Science of the Total Environment.

[ref-71] Yin Z (2002). Experimental study of assimilate production, Partitioning and Translocation among Plant Organs in Summer Maize (*Zea mays* L.) under Various Environmental and Management Conditions. Acta Agronomica Sinica.

[ref-72] Yin W, Chen GP, Chai Q, Zhao C, Feng XF, Yu QZ, Hu FL, Guo Y (2016). Responses of soil water and temperature to previous wheat straw treatments in plastic film mulching maize field at hexi corridor. Scientia Agricultura Sinica.

[ref-73] Zhai Z, Li Y, Guo J, Wang J, Pang H (2017). Effect of tillage depth on soil physical properties and yield of winter wheat-summer maize. Transactions of the Chinese Society of Agricultural Engineering.

[ref-74] Zhang S, Chen X, Jia S, Liang A, Zhang X, Yang X, Wei S, Sun B, Huang D, Zhou G (2015). The potential mechanism of long-term conservation tillage effects on maize yield in the black soil of Northeast China. Soil & Tillage Research.

[ref-75] Zhang HL, Gao WS (2005). Prospects and present situation of conservation tillage. Journal of China Agricultural University.

[ref-76] Zhang J, Wei Y, Liu J, Yuan J, Liang Y, Ren J, Cai H (2019). Effects of maize straw and its biochar application on organic and humic carbon in water-stable aggregates of a Mollisol in Northeast China: a five-year field experiment. Soil & Tillage Research.

[ref-77] Zhang Y, Wang R, Wang S, Wang H, Xu Z, Jia G, Wang XL, Li J (2017). Effects of different sub-soiling frequencies incorporated into no-tillage systems on soil properties and crop yield in dryland wheat-maize rotation system. Field Crops Research.

